# Real-time artificial intelligence-based needle tracking for ultrasound-guided regional anesthesia training: a pilot prospective randomized controlled trial

**DOI:** 10.1186/s12871-026-03867-z

**Published:** 2026-05-06

**Authors:** Misato Kurota, Kai Kubota, Tatsuya Hayasaka, Hiroaki Toyama

**Affiliations:** 1 Department of Anesthesiology, Yamagata University Hospital, 2-2-2 Iida-nishi, Yamagata, 990-9585 Japan; 2 School of Medicine, Yamagata University, Yamagata, Japan

**Keywords:** Ultrasound-guided regional anesthesia, Medical education, Artificial intelligence, Deep learning, YOLOv5, Simulation training, Needle visualization, Objective assessment

## Abstract

**Background:**

Proper needle visualization is a major technical challenge for novices learning ultrasound-guided regional anesthesia (UGRA). We developed a ‘You Only Look Once version 5’ (YOLOv5)-based system that records quantitative needle trajectory data and delivers real-time visual and auditory feedback to support training. This pilot study aimed to explore whether short-term use of the real-time feedback system improved puncture safety and whether needle oscillation amplitude, a system-derived trajectory-based metric, could serve as an objective indicator of technical proficiency in UGRA training.

**Methods:**

Twenty-three medical students with no UGRA experience were randomized into control (*n* = 12) and YOLOv5 feedback (*n* = 11) groups. Participants performed three in-plane punctures of increasing difficulty on phantoms: sagittal (puncture A), coronal (puncture B), and transverse (puncture C). Each included watching an instructional video, a 5-minute practice (with feedback for the YOLOv5 group or without for the control group), and a test puncture without feedback. The outcomes were needle tip disappearance frequency compared between the control and YOLOv5 feedback groups and needle oscillation amplitude — the maximum lateral deviation from the ideal insertion line, automatically recorded by the system — compared between punctures classified as safe and unsafe by an independent blinded assessor.

**Results:**

Needle tip disappearance frequency did not differ between the control and YOLOv5 feedback groups across all approaches (puncture A: median, 0 vs. 0, *P* = 0.933; puncture B: 0 vs. 1, *P* = 0.246; puncture C: 1 vs. 2, *P* = 0.463). Only puncture C yielded both safe and unsafe punctures, enabling comparative analysis of the needle oscillation amplitude. Participants performing safe punctures (Level 1, *n* = 9) exhibited significantly smaller needle oscillation amplitudes (136.0 ± 32.1 pixels) than those performing unsafe punctures (Level 3, *n* = 12, 230.5 ± 85.4 pixels; *P* = 0.003).

**Conclusions:**

This pilot study identified needle oscillation amplitude as a candidate objective proficiency metric in UGRA training, with preliminary evidence differentiating safe from unsafe punctures. Short-term training with the current prototype did not produce measurable improvements in puncture safety. However, the usability challenges encountered with this feedback system provided foundational insights for developing an optimized trajectory-based feedback system.

**Trial registration:**

UMIN Clinical Trials Registry (UMIN000055602), registered October 1, 2024.

## Background

Proper needle visualization is among the most significant technical challenges encountered by novices in ultrasound-guided regional anesthesia (UGRA). Maintaining continuous visualization of the needle tip during advancement toward the target is technically demanding [1]. Maintaining the needle’s alignment within the ultrasound beam plane is critical for effective analgesia and minimizing the risk of serious complications. Mastery of this technique may require approximately 28 puncture attempts under instructor feedback [2].

Recently, simulation-based education using immersive simulators and phantoms has become increasingly widespread and has been associated with improved learning efficiency and procedural accuracy [3]. Within simulation-based educational programs, feedback is a fundamental component of skill acquisition. Effective feedback promotes skill acquisition by clearly indicating the gap between the current performance and the ideal standard [4]. However, a systematic review of UGRA simulation education [5] reported that, despite widespread recognition of the importance of feedback, empirical studies evaluating its effectiveness remained limited. Several feedback devices have been developed to assist trainees in achieving accurate needle-beam alignment. Tsui developed a laser-based guidance device and proposed its potential utility for training novices in needle-beam alignment [6]. Another training system with multi-angle visualization of hand positioning demonstrated significant improvement in needle insertion accuracy among novice operators [7]. While, these devices assist only with static needle-beam alignment and do not record or provide feedback on dynamic needle trajectory information. A system that combines real-time quantitative needle trajectory recording with simultaneous visual and auditory feedback has not been widely described in the context of UGRA training. Consequently, the optimal design parameters for trajectory-based feedback remain to be established.

Beyond feedback design, the assessment of technical proficiency in UGRA training has traditionally relied on metrics such as needle tip disappearance frequency and the number of attempts required for successful puncture [1, 2]. However, these indicators capture only procedural success or failure and the presence or absence of visualization, without providing a quantitative assessment of needle trajectory. Currently, no established method allows continuous measurement of needle stability or deviation from the ideal insertion path. Quantitative evaluation of needle trajectory patterns may provide deeper insights into the process of skill acquisition.

Recently, the object detection algorithm—You Only Look Once (YOLO) [8]—has been applied to medical image analysis and surgical support systems [9], demonstrating high accuracy in real-time object tracking. We developed a YOLO-based needle tracking system designed to address the challenge of maintaining proper needle visualization among novices. The system acquires needle position coordinates in real-time to record needle trajectories and provides immediate visual and auditory feedback when deviations from the ideal insertion path occur. Unlike conventional methods that rely solely on visual feedback [6, 7], this system combines visual and auditory feedback with quantitative trajectory data, enabling puncture pattern analysis and objective assessment.

As the first implementation of a system combining real-time quantitative needle trajectory recording with simultaneous visual and auditory feedback in UGRA training, this pilot study had two objectives: (1) to preliminarily examine whether short-term training with this system improves puncture safety, and (2) to explore the utility of needle oscillation amplitude as a novel objective metric for assessing technical proficiency in UGRA training.

## Methods

### Study design and setting

This single-center, prospective, pilot randomized controlled trial was conducted at Yamagata University Hospital between November 1 and November 30, 2024. The study was conducted in accordance with the principles of the Declaration of Helsinki. The research protocol and informed consent documents were approved by the Ethics Committee of the Yamagata University School of Medicine (approval number: 2024 − 225), and the trial was registered with the University Hospital Medical Information Network (UMIN) (UMIN000055602). This study was conducted and reported in accordance with the CONSORT guidelines.

### Participants

The study enrolled medical students with no prior experience in UGRA. Written informed consent was obtained from all participants. Exclusion criteria included inability to provide informed consent and prior experience with ultrasound-guided needle procedures. The participants were allocated to either a control group trained without feedback or a You Only Look Once version 5 (YOLOv5) group (Y group) trained with feedback from the real-time feedback system. Group allocation was performed using computer-generated random numbers with a block size of four. An independent research assistant ensured allocation concealment.

Observer bias was minimized by automatically calculating needle oscillation amplitude from needle coordinate data using the YOLOv5 system.

### Devices

**Fig. 1:**
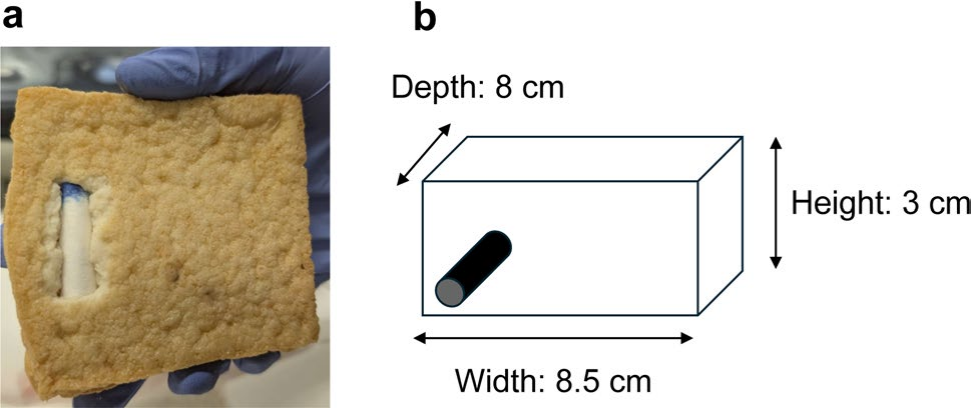
Puncture phantom. (**a**) Photograph of the tofu-based phantom and embedded target. (**b**) Schematic diagram showing the phantom dimensions (width: 8.5 cm, depth: 8 cm, height: 3 cm) with a cylindrical target (φ1 cm × 3 cm) embedded at the bottom

All procedures were performed using a GE LOGIQ e ultrasound system (GE Healthcare, Wauwatosa, WI, USA) equipped with a 9 L linear probe. A 20-gauge, 100-mm needle (Ultraplex^®^360, B. Braun Melsungen AG, Melsungen, Germany) was used for puncture. The puncture phantom was a handmade tofu-based model measuring 8.5 cm × 8 cm × 3 cm, containing a cylindrical target (1 cm diameter × 3 cm length) embedded at its base (Fig. 1).

**Fig. 2:**
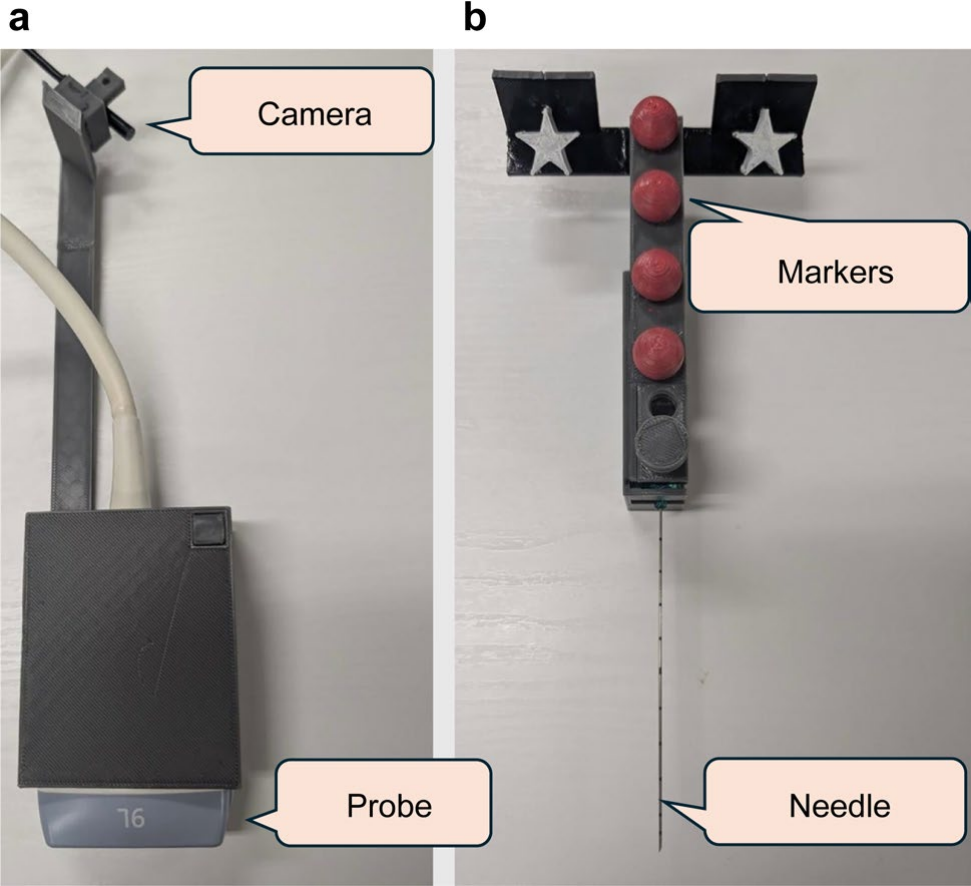
System components. Camera attached to ultrasound probe (**a**). (**b**) Puncture needle with six markers: two star-shaped and four circular for real-time positional detection

**Fig. 3:**
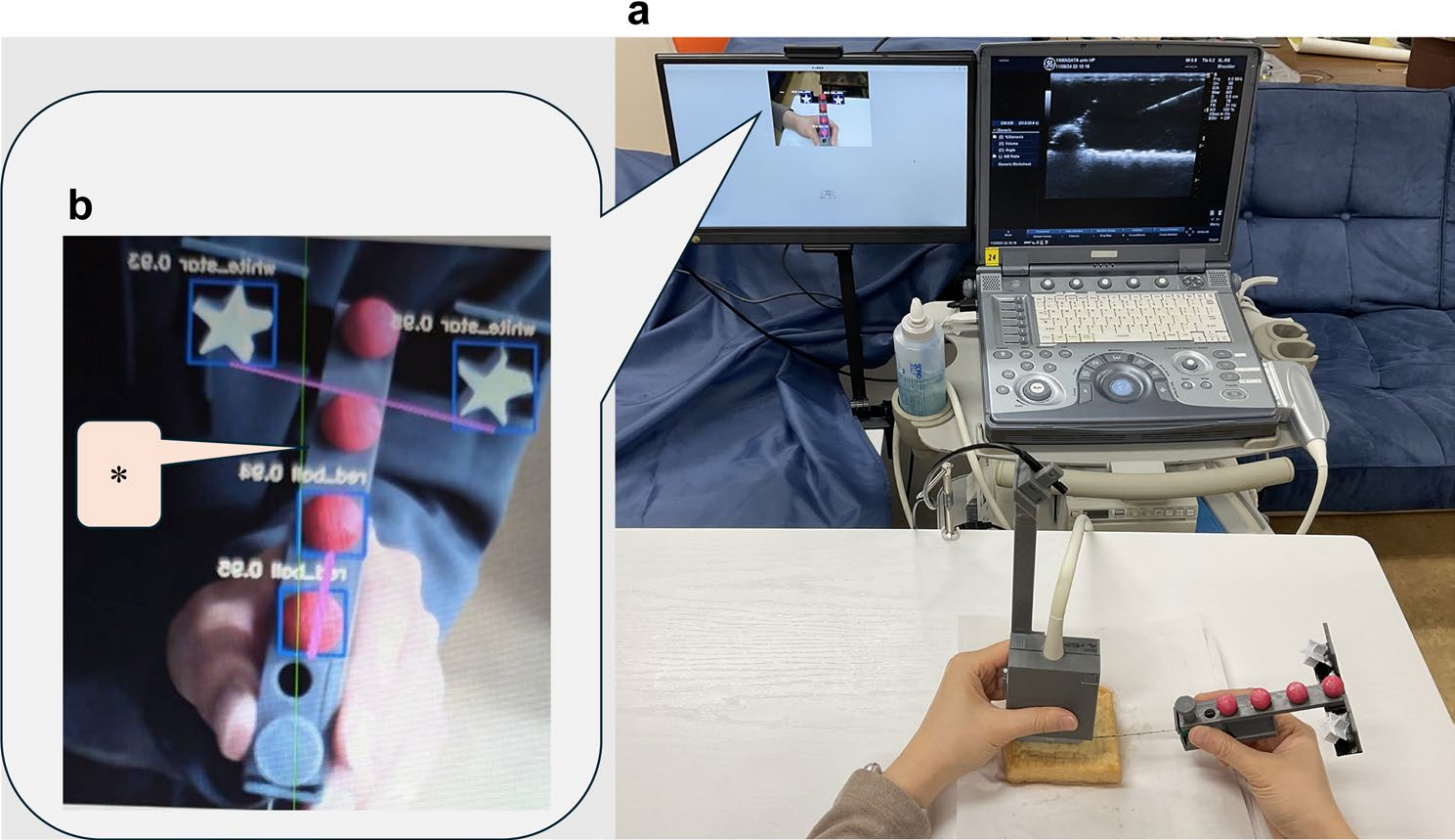
Training setup. Real-time feedback system during training (**a**). Panel **b** shows a magnified view of the feedback screen displaying detected needle markers and the ideal insertion line (＊)

**Fig. 4:**
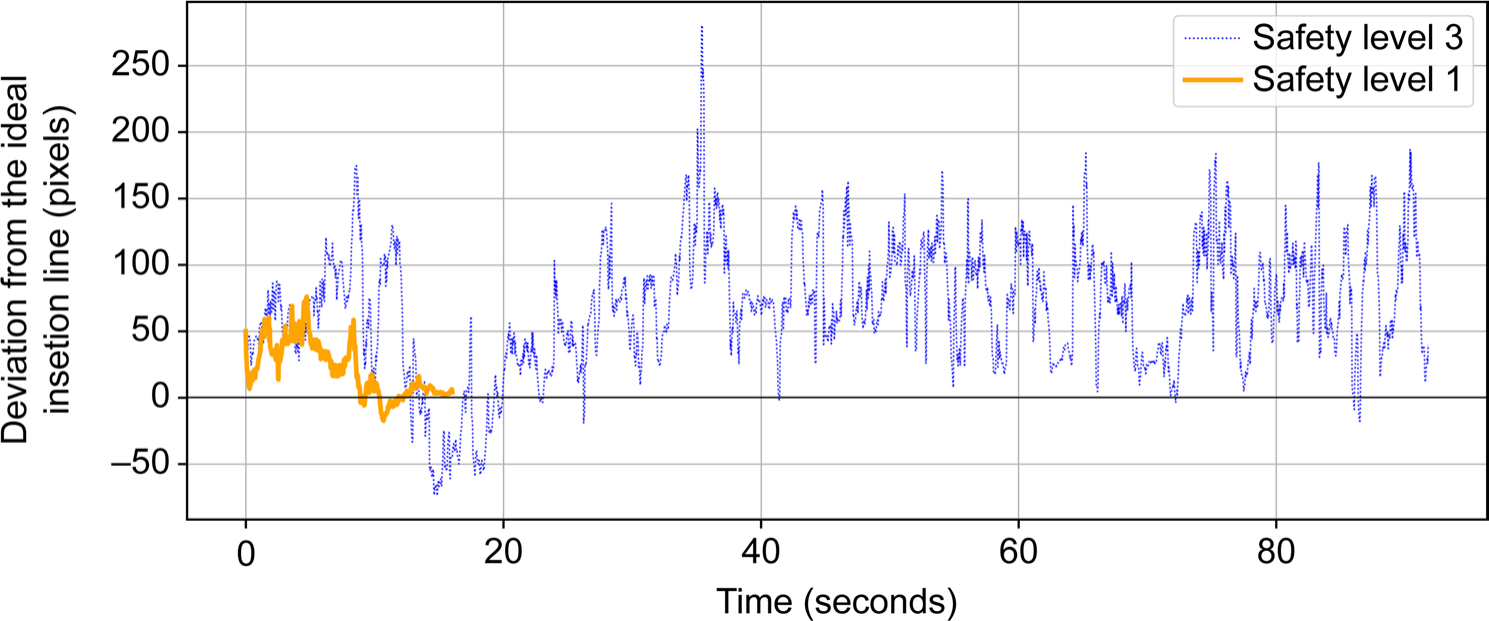
Examples of needle trajectory patterns from Level 1 and Level 3 puncture. Dotted lines indicate deviation from the ideal needle insertion line for each individual over time. The y-axis indicates the distance from the ideal insertion line in pixels. The x-axis represents the elapsed time (s). The orange solid line shows the actual trajectory data from a participant who achieved level 1 (safe) puncture, demonstrating minimal deviation from the ideal insertion line with stable and controlled progression. The dotted blue line indicates that a participant performing a Level 3 (unsafe) puncture exhibited marked oscillatory behavior with large amplitude deviations throughout the procedure

The real-time feedback system for UGRA training consists of a camera mounted on the ultrasound probe (Fig. 2a) and six markers—two star-shaped and four circular—attached to the puncture needle (Fig. 2b). The camera detects the markers on the needle to determine the needle insertion trajectory. As shown in Fig. 3, operators can monitor needle deviation in real-time by viewing a display that shows the needle image from the camera’s perspective and the ideal insertion line, defined as the center of the ultrasound probe. This system has two primary functions: a feedback system that provides real-time visual and auditory cues, and a recording function that captures needle position over time during the puncture. When the needle deviates from the ideal insertion line, an alarm is emitted to provide auditory feedback. Specifically, the system selects the two markers with the highest detection confidence from among the four circular markers positioned along the needle axis, and an auditory alert is triggered when the line connecting these two markers deviates from the ideal insertion line. The distance, measured in pixels, between the midpoint of these two markers and the ideal insertion line is plotted over time (Fig. 4). This approach enables quantitative evaluation of the needle trajectory during puncture. Marker recognition was performed using YOLOv5, a deep-learning-based object detection model. Two independent models were constructed to determine the shape of each marker. Images of needle-side markers captured by the probe-side camera were annotated to create a retraining dataset. A single researcher with expertise in this technique performed annotations using a standardized protocol to ensure consistent inclusion of all markers. In total, 215 images were annotated for star-shaped marker detection and 328 for circular marker detection. Subsequent fine-tuning was performed using the annotated dataset based on a pretrained YOLOv5 model. Marker detection performance was evaluated using an independent test dataset. The star-shaped marker detection model achieved a precision of 99.9%, a recall of 100%, and a mean average precision at an IoU threshold of 0.5 (mAP@0.5; a standard metric evaluating detection accuracy by combining localization and classification performance) of 99.5%. The circular marker detection model achieved a precision of 93.9%, a recall of 100%, and a mAP@0.5 of 99.5%. Both models operated at an average of 30 frames per second (fps), providing sufficient processing speed for real-time feedback. All punctures were performed under controlled conditions, with the light environment and the position of the camera mounted on the ultrasound probe kept constant. By combining these standardized conditions with the high-performance marker detection model, marker detection conditions were unified as much as possible for identical needle insertion angles and depths.

### Study protocol

**Fig. 5:**
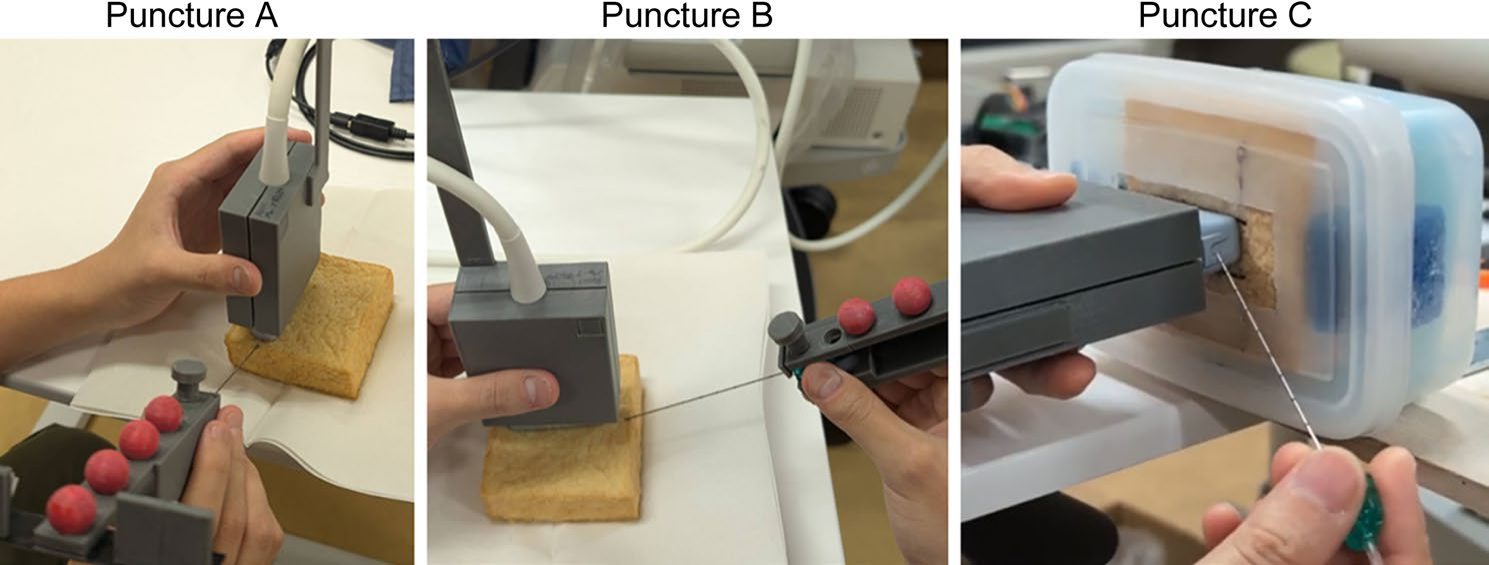
Puncture techniques. Three in-plane puncture techniques, **A**, **B**, and **C**, were performed in the following order: needle placement in the sagittal plane of the operator (puncture **A**), needle placement in the coronal plane of the operator (puncture **B**), and needle placement in the transverse plane of the needle performer (puncture **C**)

**Fig. 6:**
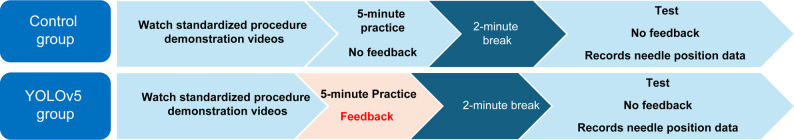
Study protocol. The control group practiced without feedback, while the YOLOv5 group practiced with real-time feedback. Both groups were tested without feedback after a 5-minute interval

The three types of in-plane puncture techniques, A, B, and C, were performed in the following order: needle placement in the sagittal plane of the operator (puncture A), needle placement in the coronal plane of the operator (puncture B), and needle placement in the transverse plane of the needle performer (puncture C) (Fig. 5). The three puncture techniques were selected to represent progressive levels of technical difficulty commonly recognized in clinical ultrasound-guided regional anesthesia practice. The techniques differ in the degree of spatial coordination required between the operator’s line of sight, needle-holding hand, and probe-holding hand, with complexity increasing progressively from Puncture A to C. For each puncture technique, the participants first watched a standardized instructional video, followed by a 5-minute practice session. During the practice, the participants were free to repeat the punctures as many times as they wished, with no limit on the number of attempts. The control group practiced without feedback, whereas the Y group practiced with real-time feedback provided by the developed training system. The researchers did not provide technical advice or verbal feedback to either group. After the practice session, both groups performed the test without feedback, and needle position information and ultrasound images were recorded. Participants were instructed to puncture the phantom and reach the embedded target within a 5-minute time limit. The procedure ended when the participant declared successful target contact or when the 5-minute time limit was reached (Fig. 6).

Two primary outcomes were defined, corresponding to our dual research objectives: (1) needle tip disappearance frequency (to assess training effectiveness) and (2) needle oscillation amplitude (to evaluate the utility of quantitative trajectory analysis in distinguishing between safe and unsafe techniques). The needle tip disappearance frequency was defined as the number of times the needle tip disappeared from the ultrasound image, and was compared between the two groups. The needle oscillation amplitude was defined as the maximum displacement amplitude in the y-axis (left-right direction perpendicular to the ultrasound beam) of the needle coordinates automatically recorded by the YOLOv5 system. Specifically, it was calculated as the difference between the maximum and minimum y-axis values among all coordinate points plotted during the puncture. An independent evaluator classified the safety of each puncture into three levels to examine the relationship between needle oscillation amplitude and puncture safety:

- Level 1 (safe): The needle tip was constantly visible and reliably reached the target, with no wandering or penetration outside the target.
- Level 2 (moderate risk): Temporary needle tip disappearance; however, the needle ultimately reaches the target, and temporary needle wandering is corrected.
- Level 3 (unsafe): Frequent or prolonged needle tip disappearance, with a clear puncture outside the target, needle wandering, or multiple reinsertions.

Based on this classification, we compared needle oscillation amplitudes between Level 1 and Level 3 punctures. We compared the distribution of puncture safety levels between the two groups.

The outcome assessment was independently performed by a single anesthesiologist (12 years of UGRA experience) who was blinded to the group allocation. Prior to study initiation, the assessor underwent training using eight separate puncture videos (unrelated to this study) to standardize the assessment criteria (three-level classification of puncture safety and determination of needle-tip disappearance frequency). The evaluation was performed using recorded ultrasound videos, with the evaluator blinded to the participant group allocation and personal information.

### Statistical analysis

Data normality was confirmed using the Shapiro–Wilk test. Intergroup comparisons were performed using the Mann–Whitney U test and t-tests. Categorical variables were compared using Fisher’s exact test. As punctures A, B, and C are technically distinct and independent procedures, intergroup comparisons for each puncture technique were treated as independent research hypotheses. Therefore, adjustments were not made for multiple comparisons. The significance level was set at *p* < 0.05. Effect sizes were reported as rank-biserial correlation (*r*), Cramér’s *V*, and Cohen’s *d* for Mann–Whitney U tests, Fisher’s exact tests, and t-tests, respectively, with 95% confidence intervals. Statistical analyses were performed using EZR software (Saitama Medical Center, Jichi Medical University, Saitama, Japan) and Python 3.12.13 (SciPy 1.16.3, NumPy 2.0.2).

As a pilot study, the enrollment target was set at 12 participants per group (24 participants in total). This target was determined based on the sample size of a similar preliminary study [10] of UGRA training. No interim analysis or discontinuation criteria were specified in advance.

## Results

**Fig. 7:**
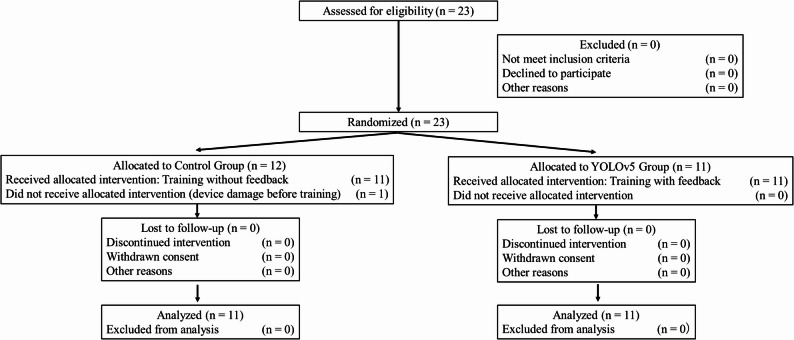
Study flow diagram. Twenty-three participants were randomized (control, *n* = 12; YOLOv5: *n* = 11). One control participant was excluded because of an equipment malfunction. Final analysis: *n* = 22 (control: 11; YOLOv5:11)

Twenty-three participants were randomly allocated to either the control group (*n* = 12) or the Y group (*n* = 11). One participant in the control group was excluded before training because of equipment malfunction resulting from accidental device damage, which precluded participation. The final analysis included 22 participants: 11 and 11 in the control and Y groups, respectively (Fig. 7). The median age was 23 years in both groups. The control group included eight males and four females, whereas the Y group included five males and six females.

Needle tip disappearance frequency, showed no significant differences between the groups (Mann–Whitney U test) for puncture A (control group vs. Y group: median 0 vs. 0 times, *P* = 0.933, *r* = 0.025, 95% CI [-0.364, 0.397]), puncture B (median 0 vs. 1 time, *P* = 0.246, *r* = 0.273, 95% CI [-0.165, 0.669]), or puncture C (median 1 vs. 2 times, *P* = 0.463, *r* = 0.190, 95% CI [-0.322, 0.669]). In the blinded safety assessment, the distribution of safety levels showed no statistically significant differences between the two groups for any puncture method (Puncture A, *P* = 1.000, V = 0.218, 95% CI [0.218, 0.397]; Puncture B, *P* = 0.311, V = 0.325, 95% CI [0, 0.647]; Puncture C, *P* = 1.000, V = 0.225, 95% CI [0.221,0.581]; Fisher’s exact test). No participants in either group were classified as Level 3 (unsafe) for punctures A and B. For the most difficult puncture (C), 55% (6/11) of the control group and 55% (6/11) of the Y group were classified as Level 3, suggesting a possible relationship between puncture difficulty and safety performance, regardless of the training method (Table 1).

**Table 1:** Disappearance frequency and safety outcomes across three puncture techniques

	Control group (*n* = 11)	YOLOv5 group (*n* = 11)	*P* value	Effect size *r* (95%CI)
Puncture A				
Needle tip disappearance frequency	0 [0–1]	0 [0–5]	0.933	0.025
Puncture safety level, *n* (%)				(-0.364, 0.397) Effect size V (95%CI)
Lv.1	11 (100%)	10 (91%)	1	0.218
Lv.2	0 (0%)	1 (9%)		(0.218, 0.397)
Lv.3	0 (0%)	0 (0%)		
Puncture B				Effect size r (95%CI)
Needle tip disappearance frequency	0 [0–2]	1 [0–4]	0.246	
Puncture safety level n (%)				0.273 (-0.165, 0.669) Effect size V (95%CI)
Lv.1	10 (91%)	7 (64%)	0.311	0.325
Lv.2	1 (9%)	4 (36%)		(0, 0.647)
Lv.3	0 (0%)	0 (0%)		
Puncture C				Effect size r (95%CI)
Needle tip disappearance frequency	1 [0–12]	2 [0–7]	0.463	0.190
Puncture safety level n (%)				(-0.322, 0.669) Effect size V (95%CI)
Lv.1	5 (45%)	4 (36%)	1	0.225
Lv.2	0 (0%)	1 (9%)		(0.221, 0.581)
Lv.3	6 (55%)	6 (55%)		

Data are presented as median [range] for needle tip disappearance frequency and number of cases for safety levels (%). P-values calculated using the Mann–Whitney U test (disappearance frequency) and Fisher’s exact test (safety level distribution)

We analyzed needle oscillation amplitude during puncture C to identify the quantitative characteristics distinguishing safe from unsafe techniques, as unsafe techniques (Level 3) occurred exclusively during this condition. Participants performing puncture C were categorized into Level 1 (*n* = 9) and Level 3 (*n* = 12) groups based on a blinded safety assessment. The mean needle oscillation amplitude in the Level 3 group (230.5 ± 85.4 pixels) was significantly greater than that in the Level 1 group (136.0 ± 32.1 pixels) (*P* = 0.003, 95% CI of difference: 37.2-151.8 pixels, Cohen’s d = 1.385, t-test) (Table 2).

**Table 2:** Comparison of needle oscillation amplitude by safety level in Puncture C

	Safety Level				
	Level 1 (Safe) (*n* = 9)	Level 3 (Unsafe) (*n* = 12)	*P* value	Mean difference (95%CI)	Cohen’s d (95%CI)
Needle oscillation amplitude (pixels)	136.0 ± 32.1	230.5 ± 85.4	0.003	94.5 (37.17, 151.83)	1.385 (0.402, 2.339)

Data are presented as mean ± SD

Figure 4 shows the actual needle trajectory patterns from two participants. Trajectory data from participants who achieved Level 1 (safe) puncture demonstrated stable and controlled progression, with minimal deviation from the ideal insertion path. In contrast, participants performing Level 3 (unsafe) punctures exhibited marked oscillatory behavior with large-amplitude deviations throughout the procedure. Individual trajectory patterns visually confirmed the quantitative differences in needle oscillation amplitudes reported in Table 2, supporting the objective distinction between safe and unsafe puncture techniques.

## Discussion

This pilot study had two objectives. First, as a preliminary examination of educational effectiveness, short-term training with the real-time feedback system did not produce measurable improvements in puncture safety in this initial implementation of the prototype. Second, we proposed needle oscillation amplitude as a novel objective metric for assessing technical proficiency in UGRA training. This quantitative trajectory parameter successfully distinguished between safe and unsafe puncture techniques, with a large effect size (Cohen’s d = 1.385).

The preliminary examination of educational effectiveness revealed two important insights to inform the future development of trajectory-based feedback systems. First, regarding feedback design, no prior evidence existed to guide the optimal design of visual information display or the timing and threshold of auditory feedback for this novel system. The auditory feedback was designed to trigger an alarm whenever the needle deviated from the ideal insertion line. However, this design resulted in frequent alarms even when the needle remained visible on the ultrasound image, causing confusion and distraction among participants. Additionally, the feedback display presented the needle image from the opposite perspective of the operator, resulting in a left-right reversal that made it difficult to intuitively determine the direction of needle deviation. Slater et al. [4] emphasized that targeted feedback is critical as the capacity to accept and use feedback is limited. The usability challenges identified in this study suggest that alarm threshold calibration and intuitive visual display design are essential requirements for effective trajectory-based feedback. Second, regarding the target learner population, the effectiveness of feedback varies depending on the learner’s proficiency level [4]. The participants were considered to be in the cognitive stage of motor skill acquisition, in which processing complex feedback information while simultaneously interpreting ultrasound images, manipulating the needle, and holding the probe may impose excessive cognitive load. It has also been noted that concurrent feedback may be particularly beneficial once learners have acquired a basic understanding of the task [4]. The cognitive overload observed in this study suggests that early learners with basic UGRA knowledge, rather than complete novices, may represent a more appropriate target population for this real-time trajectory-based feedback system.

In the proficiency assessment of UGRA training, needle tip disappearance frequency and puncture success rate are the primary indicators traditionally used [1, 2]. However, no quantitative indicators have been established to evaluate the stability and accuracy of needle manipulation, which are important characteristics for safe puncture techniques. In this study, we continuously recorded needle trajectories and quantified the lateral displacement from the ideal insertion line (needle oscillation amplitude) to investigate whether this metric could serve as an objective indicator of proficiency in puncture techniques. Our study demonstrated that the participants who underwent safe insertions had significantly smaller needle oscillation amplitudes than those who underwent unsafe insertions. Motor learning theory suggests that as skills develop, movement becomes less redundant and more efficient. This finding is consistent with the study by Sites et al. [1], which reported excessive unnecessary movements in UGRA novices, suggesting that needle oscillation amplitude may serve as an indicator of inadequate technical proficiency. The large effect size observed (Cohen’s d = 1.385) further supports the robustness of this metric, despite the limited sample size of this pilot study.

Several objective assessment methods have been reported for UGRA training, including quantitative hand motion analysis [11] and eye tracking systems [12]. Although hand movement analysis systems can comprehensively evaluate hand movements, directly assessing the clinically critical indicator of deviation from the ideal insertion path is challenging. Eye-tracking systems are effective for evaluating cognitive processes such as gaze patterns and fixation behavior. However, because visual attention and motor performance are fundamentally different processes, eye-tracking systems have limitations in assessing needle manipulation accuracy and puncture technique precision. Unlike existing evaluation methods, our system quantitatively records the deviations from the ideal insertion path. The needle oscillation amplitude measured using our system reflects the variation in the position of the needle relative to the probe. Because UGRA requires coordinated manipulation to maintain the needle within the ultrasound beam while fine-tuning the probe, this metric is clinically valid for assessing needle-probe coordination ability.

Although real-time feedback did not produce measurable improvements in complete novices in this study, the findings do not preclude the potential educational value of this system. Future studies should examine whether longer and repeated training using an optimized feedback system leads to measurable improvements in puncture safety among early learners with basic UGRA knowledge. Furthermore, the needle trajectory recorded by this system can also be utilized as a source of post-hoc feedback. Unlike real-time feedback, post-hoc feedback allows even complete novices with no prior UGRA experience to reflect on their own performance in a less cognitively demanding context.

Regarding needle oscillation amplitude as a novel proficiency metric, future studies should collect data from larger and more diverse trainee populations, including those with varying levels of UGRA experience, to characterize puncture patterns according to proficiency level. By comparing individual trainees’ puncture patterns against proficiency-based reference data, specific technical weaknesses may be identified, potentially facilitating the development of personalized educational programs. Furthermore, longitudinal measurement of oscillation amplitude throughout the training process may capture temporal changes in skill acquisition, potentially providing a more precise basis for structuring training programs.

This study has some limitations. First, because the pilot study was conducted with a limited sample size, it may have lacked sufficient statistical power. Therefore, the results should be interpreted as exploratory findings and require validation in future large-scale studies. Second, because the participants were limited to medical students, the results may not be generalizable to all UGRA novices. Third, because only one evaluator performed the puncture safety assessment, inter-evaluator reliability was not verified. Fourth, because this study used pixels to evaluate needle oscillation amplitude, its clinical significance is unclear. The camera was not calibrated to convert pixel measurements to millimeters. Therefore, we cannot definitively determine the actual distance (in millimeters or centimeters) from the ideal insertion line. Future studies should properly calibrate the camera system using an object of known dimensions so that pixel measurements can be converted into clinically meaningful distance units. Despite this limitation, the relative difference in the oscillation amplitude between safe and unsafe punctures (approximately 1.7 times) provides meaningful comparative data for skill assessment. Fifth, since the system selects the two markers with the highest detection confidence from among the four circular markers, the midpoint position may vary depending on which markers are selected. This could potentially affect the measured distance, particularly when the needle significantly deviates obliquely from the ideal insertion line. Although punctures were performed under standardized conditions to minimize variability in marker detection, quantitative verification of the stability of marker selection remains a subject for future investigation. Sixth, this study did not record the number of puncture attempts during the practice session. Future studies should systematically record practice attempts and optimize the system design simultaneously to more rigorously evaluate the educational potential of trajectory-based feedback.

## Conclusions

This pilot study provided preliminary evidence that needle oscillation amplitude has potential as a novel objective metric for distinguishing between safe and unsafe puncture techniques, supported by a large effect size (Cohen’s d = 1.385). Regarding educational effectiveness, short-term training with the current prototype did not produce measurable improvements in puncture safety. This finding, combined with the usability challenges identified in this initial implementation, highlights the importance of optimizing feedback design and carefully defining the target learner population in future studies. Collectively, these findings provide a foundation for the development of an optimized trajectory-based feedback system.

## Data Availability

The datasets used and/or analyzed in the current study are available from the corresponding author upon reasonable request.

## Abbreviations

UGRA: Ultrasound-guided Regional Anesthesia
YOLO: You Only Look Once
YOLOv5: You Only Look Once version 5
mAP@0.5: Mean Average Precision at intersection over union threshold of 0.5
